# A 12-week multidomain intervention versus active control to reduce risk of Alzheimer’s disease: study protocol for a randomized controlled trial

**DOI:** 10.1186/1745-6215-14-60

**Published:** 2013-02-27

**Authors:** Kaarin J Anstey, Alex Bahar-Fuchs, Pushpani Herath, George W Rebok, Nicolas Cherbuin

**Affiliations:** 1 Centre for Research on Ageing Health and Wellbeing, The Australian National University, Canberra, Australia; 2 Department of Mental Health, and the Center on Aging and Health, The Johns Hopkins University, Baltimore, MD, USA

**Keywords:** RCT, Alzheimer’s disease, Behavior change, Internet, Physical activity, Diet, Cardiovascular risk factors

## Abstract

**Background:**

Disappointing results from clinical trials of disease-modifying interventions for Alzheimer’s dementia (AD), along with reliable identification of modifiable risk factors in mid life from epidemiological studies, have contributed to calls to invest in risk-reduction interventions. It is also well known that AD-related pathological processes begin more than a decade before the development of clinical signs. These observations suggest that lifestyle interventions might be most effective when targeting non-symptomatic adults at risk of AD. To date, however, the few dementia risk-reduction programs available have targeted individual risk factors and/or were restricted to clinical settings. The current study describes the development of an evidence-based, theoretically-driven multidomain intervention to reduce AD risk in adults at risk.

**Method:**

The design of Body Brain Life (BBL) is a randomized controlled trial (RCT) to evaluate a 12-week online AD risk-reduction intervention. Eligible participants with several modifiable risk factors on the Australian National University (ANU) AD Risk Index (ANU-ADRI) are randomly allocated to an online only group, an online and face-to-face group, or an active control group. We aim to recruit 180 participants, to undergo a comprehensive cognitive and physical assessment at baseline, post-intervention, and 6-month follow-up assessment. The intervention comprises seven online modules (dementia literacy, risk factor education, engagement in physical, social, and cognitive lifestyles, nutrition, and health monitoring) designed using contemporary models of health behavior change.

**Discussion:**

The BBL program is a novel online intervention to reduce the risk of AD in middle-aged adults at risk. The trial is currently under way. It is hypothesized that participants in the intervention arms will make lifestyle changes in several domains, and that this will lead to a reduction in their AD risk profile. We also expect to show that health behavior change is underpinned by changes in psychological determinants of behavior. If successful, the findings will contribute to the development of further dementia risk reduction interventions, and thus contribute to the urgent need to lower dementia risk factors in the population to alter future projections of disease prevalence. Longer follow-up of BBL participants and replications using large samples are required to examine whether reduction in AD risk factors will be associated with reduced prevalence.

**Trial registration:**

Reg. no. ACTRN12612000147886

## Background

As the most common cause of dementia, Alzheimer’s disease (AD) affects approximately 35 million people worldwide, and this will increase with population ageing [1]. There is increasing focus on delaying the onset of AD through intervening to modify risk factors [2,3], and it has recently been estimated that a 10% to 25% reduction in 7 key risk factors could prevent 1.3 million AD cases internationally [2]. To our knowledge only a few prevention trials for Alzheimer’s disease are currently under way, and interventions that could be delivered at the population level (as opposed to interventions conducted in clinical settings) have not yet been developed. The largest ongoing trial of dementia prevention is the Multi-domain Alzheimer Prevention Trial (MAPT), which involves MRIs, fish oil and clinical assessments, and costs millions of dollars. Other randomized controlled trials (RCTs) targeting dementia prevention have either focused on a single risk factor (for example, hypertension) or are clinically based and require full medical assessments and expensive treatment regimes. There is an urgent need to develop population-level, low-cost interventions that will reduce the risk of AD.

The present study builds on our research program on dementia risk reduction that commenced in 2005 and has led to four systematic reviews [4-7], and the development of evidence-based and validated self-report tool for the assessment of risk of AD [8]. We have also examined predictors of conversion to preclinical dementia in our PATH Project [9]. We have designed an intervention study to reduce the risk of AD by concurrently targeting modifiable risk factors in middle-aged adults. The intervention is designed for distribution via the Internet, and hence, may reach a far wider group of individuals within the population than the high-cost medical interventions currently under way.

Risk factors for cardiovascular disease in older persons are well established and include several modifiable lifestyle factors. More recently, converging evidence from large epidemiological studies has confirmed that some of the central modifiable risk factors for cardiovascular disease are also risk factors for AD, the most common cause of dementia in people over 65 years of age. These factors include smoking, alcohol consumption, poor diet, depression, and physical inactivity. Other factors that appear to play a protective role in the prevention of AD, such as engagement in social and cognitively stimulating lifestyles, may play less of a role in the prevention of cardiovascular disease. The proposed trial (reg. no. ACTRN12612000147886) is currently under way in Australia.

### Targeting mid-life adults at risk

To date, most interventions to reduce dementia risk have generally targeted older individuals (>65 years), who are at risk of AD due to the presence of objective or subjective cognitive decline, a positive family history of dementia, or the detection of disease biomarkers. However, it is widely accepted now that pathological processes that lead to the development of clinical AD commence years and even decades before the onset of any detectable clinical symptoms [10]. It has been suggested that intervening at the point at which clinical symptoms become apparent may be too late. In addition, commencing in 2005, a series of systematic reviews by our group has demonstrated the links between several risk factors in mid life and the likelihood of developing dementia later in life [4-7]. Building on our findings and those of other groups, we have recently developed and validated a self-reported measure of AD risk at mid life [8]. Using such a tool in the development of interventions to reduce the prevalence of dementia risk factors in mid life may therefore have far-reaching implications for the incidence of dementia in later life.

### Use of behavior change theory

Although theory-driven interventions have been developed in the context of single risk factors to prevent cardiovascular disease (see, for example, [11]), research into dementia that concurrently targets various risk factors is in its infancy. Existing interventions have not been explicitly developed against a clear theoretical framework of behavior change. However, there is evidence that lifestyle interventions informed by theoretically-driven behavior change models are more successful and lead to stronger and more lasting changes [12]. Consequently, a complex intervention based on an emerging theoretical and empirical model of health-related behavior change [13-15] has been developed for the current project.

### Online delivery

The percentage of interventions developed and delivered over the Internet has grown dramatically over the past decade. Internet-delivered interventions are appealing to public health professionals for several reasons. First and most importantly, online interventions provide the opportunity for large-scale implementation at the population level compared with more traditional face-to-face interventions. Further, although the initial cost associated with setting up such interventions is at times relatively high, their cost effectiveness lies in the relatively small ongoing costs, which coupled with their wide reach has the potential of having large impact [16]. Finally, the ever-increasing sophistication of Internet-based health interventions enables them to become individually tailored [17]. Evidence has emerged in recent years of the effectiveness of online health interventions in changing behavior including smoking and alcohol use [18-20], and it is likely that in the years to come this will become a primary mode of intervention delivery. To our knowledge, however, there are no existing interventions to reduce the risk of AD delivered over the Internet.

## Methods

### Study setting and design

The BBL project is a 12-week, single-blind randomized control trial of a behavior change intervention targeting established risk factors for AD delivered to cognitively healthy adults aged 50 to 60 either exclusively online or as a combination of online and small group meetings. These two groups are being compared to an active control group receiving weekly email links to relevant health information. The study is being conducted in Canberra, Australia.

### Participants

Recruitment for this study is currently ongoing. Participants are being recruited from the community via advertising in local newspapers and radio, as well as by distributing fliers in community health centers, community clubs, and through word of mouth. Participants who call the study number are added to a database of those expressing an interest in the study. Potential participants are then contacted by phone or email and are provided with additional details regarding the study and with a brief set of questions to establish whether basic inclusion criteria (see below) are met. Participants are also asked at this point to consent to undergo further screening in the form of two brief phone interviews to further establish the presence of inclusion or exclusion criteria.

### Inclusion criteria

Study prerequisites are that participants are aged 50 to 60, reside in the Australian Capital Territory or surrounding areas of New South Wales, have access to a computer and Internet connection at home, and are fluent in English. To be eligible for the study, participants are required to meet a minimum of three of the following risk factors for AD: formal educational attainment at high-school level or less, sedentary lifestyle, overweight or obese body mass index (BMI), low consumption of fish, low cognitive or social engagement, as well as a history of diabetes, hypertension, high cholesterol, mild to moderate traumatic brain injury, smoking, or depression. Participants are required to be able to attend the Centre for Research on Ageing, Health, and Wellbeing at baseline and after 24 weeks for face-to-face evaluations.

### Exclusion criteria

Participants are not eligible to enroll in the trial if they have a history of neurological or psychiatric conditions likely to substantially affect cognition (for example, recent stroke, epilepsy, schizophrenia), sensory deficits or mobility limitations that would prevent or substantially restrict the delivery of the assessment or intervention (for example, uncorrected substantial loss of hearing or vision, severe physical disability), as well as other significant health problems (for example, recent cardiovascular event, renal failure, treatment for cancer). Participants are also required to obtain a score greater than 24 on the TELE instrument (a phone-based assessment of mental status) [21] to exclude the presence of global cognitive impairment.

### Phone interviews

Two brief phone interviews conducted by research assistants with graduate psychology training using a written protocol are being conducted with participants as part of the screening process. The first interview focuses on past and present medical conditions that form the study’s inclusion and exclusion criteria, while the second interview focuses on lifestyle factors. Each of the phone interviews generally lasts approximately 10 minutes.

### Sample size calculations

Sample size calculations were estimated using G*Power (version 3.1.3)(Available at: University of Dusseldorf available at http://www.psycho.uni-duesseldorf.de/aap/projects/gpower/.) and were based on medium effect sizes found for multidomain lifestyle interventions for the primary prevention of cardiovascular disease (CVD) [22] due to the lack of published trials on dementia risk reduction. To detect a medium effect in a 3-group design (1:1:1), with a 5% risk of type 1 error (α) and 80% power, a total sample size of 159 persons is required. A medium effect (0.5 standard deviation) on the Australian National University AD Risk Index (ANU-ADRI) is 4.25 points on the scale as described previously [8]. This figure is derived from the pooled standard deviation of the ANU-ADRI from three cohort studies with combined N of 4301; the Rush Memory and Aging Study, the Kungsholmen Project, and the Cardiovascular Health Study. To account for attrition, a baseline sample of 180 is being recruited.

### Assessments

Table 1 summarizes the primary and secondary assessment measures and the assessment schedule.

**Table 1:** Assessment measures at the baseline, post-intervention, and follow-up evaluations

**Assessment measure**	**Baseline**	**12 weeks**	**24 weeks**
Questionnaires			
ANU-ADRI^a^	X	X	X
Dementia literacy questionnaire^a^	X	X	X
Motivation questionnaire^a^	X	X	X
Cognitive measures			
Logical memory	X		X
RAVLT	X		X
RCFT	X		X
COWAT (FAS)	X		X
Category fluency (animals, boys, category switching)	X		X
WTAR	X		X
Digit span	X		X
Digit-symbol matching^a^	X		X
Trials A + B^a^	X		X
Physical evaluation			
Blood pressure	X		X
Height, cm	X		X
Weight, kg	X		X
Hip to waist ratio	X		X
Forced expiratory volume	X		X
Forced vital capacity	X		X
Quadriceps strength	X		X
Blood tests (optional)			
Lipids	X		X
ALT	X		X
HbA1c	X		X
Renal function	X		X

^a^Administered online on the trial website.

ALT, alanine aminotransferase; ANU-ADRI, Australian National University Alzheimer’s Disease Risk Index; COWAT, Controlled Oral Word Association Test; Hb, hemoglobin; RAVLT, Rey Auditory Verbal Learning Test; RCFT, Rey Complex Figure Test; WTAR, Wechsler Test of Adult Reading.

#### Primary outcome

The primary outcome measure is the ANU-ADRI [8]. The ANU-ADRI was developed following a synthesis of meta-analyses of various risk factors for AD reported in the literature. The questionnaire covers several modifiable risk factors and is based on self-report.

#### Secondary outcomes

Domain-specific psychological determinants questionnaires: For each intervention domain, a questionnaire was developed evaluating 14 empirically-derived theoretical determinants of behavior [23]. Changes in psychological determinants of behavior are hypothesized to underpin lifestyle changes. Domain-specific questionnaires are completed at the start of each behavior-change module, and are then used to tailor the content of online modules to the needs of individual participants. Domain-specific questionnaires will be readministered as part of the post-intervention assessment (12 weeks).

Dementia literacy [24]: this is assessed using a measure developed by the authors at the baseline, immediate post-intervention, and follow-up assessments.

Cognitive assessment: the cognitive evaluation includes the administration of several commonly used measures of cognitive ability, including measures of estimated intellectual ability, information processing speed, attention, memory, and executive function. These measures are administered as a combination of paper-and-pencil tests, and online via the trial website (Table 1).

Physical assessment: the physical evaluation includes the assessment of lung capacity measured with a spirometer (yielding forced expiratory volume and forced vital capacity), blood pressure (measured twice for increased reliability), knee extension (quadriceps strength), height, weight, and waist circumference.

Blood measures (optional): participants who consent to the blood test are referred to a central agency in Canberra (Capital Pathology) for blood collection. The following tests are requested: Fasting blood glucose, cholesterol, triglycerides, full blood count, liver function, renal function, and hemoglobin HbA1c. In addition, plasma is being separated and stored using standard procedures, in order to examine AD blood biomarkers at a later date. Participants who consent to the blood test are asked to provide the details of their general practitioner (GP), and all copies of the blood test results are forwarded to the nominated GP after being received by the study team.

### Interventions

#### Group 1: BBL online only (BBL)

Participants in the online only group log on to the trial website weekly to complete an online session lasting approximately 1 h. The 12-week program is detailed in Table 2. The first 7 weeks include the completion of seven educational and individually-tailored behavior-change modules. In the remaining 5 weeks, participants undertake online activities focused on activity and goal monitoring and revision. Tailoring of the five behavior-change modules (weeks 3 to 7) is conducted using an automated algorithm that presents content on the basis of whether or not the particular risk factor the module addresses applies to the participants, as well as on the basis of their responses to several questions measuring psychological determinants of behavior which are presented at the beginning of each of the behavior-change modules. For example, a person who is classified as sedentary on the basis of their responses on the ANU-ADRI, and who does not regard himself/herself as optimistic regarding their prospect of change in the area of physical activity, is presented with information about learned optimism and the relationship between optimism and a range of health outcomes. A physically active person who also regards himself/herself as a role model to others in the area of physical activity will not be presented with information focusing on becoming a role model to others.

**Table 2:** Description of the 12-week online program delivered through the Body Brain Life (BBL) trial website

**Week**	**Activity**	**Description**
1	Module 1: Dementia literacy	The first module focuses on providing participants with general information about dementia including types, onset, symptoms, diagnosis, progression, burden and risk factors (modifiable and non-modifiable). This module serves as an introduction to the subsequent modules.
2	Module 2: Dementia risk factors	This module is aimed at building awareness and knowledge of the various health conditions associated with an increased risk of Alzheimer’s dementia (AD). Specifically, this module provides details regarding the association between AD and several medical conditions (abnormal weight, high cholesterol, diabetes, hypertension, and depression), as well as lifestyle factors (physical activity, nutrition, social and cognitive engagement).
3	Module 3: BBL FIT - physical activity	This is a theory-driven, individually-tailored module that aims to help participants incorporate regular physical activity into their daily routine and reduce sedentary behavior. This module targets several risk factors concurrently, including sedentary lifestyle, abnormal weight, depression, and various medical conditions.
4	Module 4: BBL nutrition	This is a theory-driven, individually-tailored module aimed at helping people develop healthy dietary habits. This module targets the risk associated with abnormal weight, and the protective effects associated with fish intake and with other dietary components.
5	Module 5: BBL connect - social engagement	This is a theory-driven, individually-tailored module aimed at increasing participants’ levels of social engagement. The module targets the risk factor for dementia associated with depression, and the protective effects of regular social engagement.
6	Module 6: BBL Think - cognitive engagement	This is an individually-tailored module aimed at increasing participants’ levels of engagement with mentally stimulating activities, which is a protective factor against dementia.
7	Module 7: BBL health self-management	This is an individually-tailored module aimed at increasing participants’ health monitoring and management of chronic health conditions. Because several chronic health conditions are associated with increased risk for dementia, prevention and appropriate management of such conditions is also likely to be protective against dementia.
8 to 12	Self-guided online activities	During these sessions, participants are encouraged to engage in a range of online activities for 1 h, including accessing the many tools they have accumulated during the first 7 weeks. Examples include the goal-setting tool, behavior-monitoring tool, unhelpful thoughts monitoring tool, videos, and so on.

The program is built in such a way that participants are only able to access the relevant component of the intervention at a given time. The modules become active, one per week, on the same day for the first 7 weeks. Participants are unable to access a newly activated module before completing the previously scheduled module. Each week, participants receive a notification email on the same day alerting them when a new module has become active. The automatic notification system on the trial website is able to detect whether a given participant has not commenced, commenced but not completed, or completed a particular module, and the weekly messages are modified accordingly. Participants receive up to two automatic reminders to complete a module, and this is then followed by a text message, before phone contact is initiated by the study team. As per participation consent agreements, participants who fail to complete a module after these attempts are excluded from the study.

#### Group 2: BBL online + face-to-face (BBL + FF)

This group participates in the online program in the same way as group 1, and in addition attends five face-to-face sessions conducted in small groups facilitated by a clinical psychologist (weeks 3, 5, 7, 9 and 12). The content of the group sessions is organized around the themes of the corresponding online modules. The sessions include facilitated discussions of the various risk factors for dementia, goal setting, and barriers to behavior change. To monitor and evaluate fidelity of delivery, the sessions are pre-scripted and a subset is recorded and are subsequently analyzed.

Contact with participants in this group is similar to the contact made with participants in the online only group (that is, using the automated website notification system), with the exception that participants in this group also receive additional emails to remind them of upcoming face-to-face sessions. As there is a single interventionist (clinical psychologist) conducting the face-to-face groups, we will not address interventionist performance variability in the analysis.

#### Group 3: Active control (ACON)

The active control group does not access the trial website. Instead, participants in this group receive weekly emails containing links to health-related websites, videos, news items, and so on. The weekly emails contain several links, and participants are encouraged to spend an hour each week browsing through the material. The material is generally organized around the same themes as the ones included in the online BBL program. An effort has been made to include links to informational and educational material but that otherwise does not include the use of identifiable behavior-change techniques which are the ‘active ingredient’ of the BBL program (for example, vicarious reinforcement, anticipated regret, and so on). In addition, other than providing participants with the weekly emails, no further contact is made with the participants in this group, such as reminders and prompts that are delivered to participants in the two experimental groups.

### Randomization

A permuted block randomization sequence comprising block sizes of 30 stratified by gender is used. The allocation sequence is generated by an independent researcher following the baseline assessments and is not known to the study team at the time of enrollment and baseline assessment.

### Blinding

To prevent evaluation bias, research staff conducting the psychological, physical, and cognitive outcome assessments, as well as those involved in the analysis of pathology data remain blind to participants’ group allocation. The contact person for participants’ website queries, access issues, and technical difficulties is independent of all baseline assessment data. All participants are informed that they are being randomly allocated to one of three study groups and that one group may be more effective than others. They are also notified at the start of the study that one of these groups involves several face-to-face sessions. Hence, participants in the two online groups are kept blinded to group allocation, whereas those allocated to the online + face-to-face group are naturally able to tell the group they have been allocated to.

### Statistical analyses

Statistical analyses will be based on an intention-to-treat approach. Recent commentary on the best practice for dealing with missing data in RCTs suggests that multiple imputation and Mixed Models are effective approaches and superior to the commonly used techniques like ‘last observation carried forward’ for missing data [25,26]. We plan to use Mixed Models. We hypothesize that BBL + FF > BBL > ACON. The hypothesis that BB + FF > BBL is based on research [27] showing that social milieu and personal contact with researchers are associated with better outcomes in online interventions. There is no empirical evidence on which to base the hypothesis that BBL + FF > BBL in relation to dementia risk reduction per se, due to lack of published data. Results will be exploratory because there are no previous findings in the literature to confirm. There is random allocation to groups so we do not plan to adjust for baseline variables in the primary analysis. In sub analyses we will adjust for baseline measures such as compliance in completing the online modules (in the BBL and BBL + FF groups) by including the number of online modules completed as a fixed effect.

### Ethics

The Human Research Ethics Committee at the Australian National University has approved the study protocols and procedures.

### Adverse events

This study evaluates a lifestyle intervention program to reduce risk factors for AD. The target population is adults in mid life who have some of the known risk factors for dementia, but are at the time of the intervention healthy and free of any dementia-related symptoms. We do not anticipate that participants will be placed at a greater risk than that associated with self-driven educational activities over the Internet. Results of screening blood tests are sent to participants’ primary health care providers regardless of their group designation. Furthermore, if any abnormality is detected, a letter is sent to participants to inform them of the presence of the abnormal result and encouraging them to contact their general practitioner for medical advice. To address issues of potential fatigue, the baseline assessment has been kept to a minimum length. In addition, all online and face-to-face sessions were designed in an interactive way and are limited to 1-h sessions (90 minutes for the face-to-face groups). As mentioned above, all online modules are delivered in an individually-tailored fashion to maximize relevance for each individual.

## Discussion

The BBL project is currently under way as an evaluation of the efficacy of a novel intervention in a randomized control trial design. The design of the BBL program aimed to overcome some of the central limitations of similar intervention efforts to date by primarily using a validated AD risk assessment tool, by targeting individuals at late mid life, by developing the intervention content against sound theoretical background, and by choosing an individually-tailored, online delivery format.

The trial, advertised through local radio and newspapers, community clubs, health centers, and noticeboards, generates considerable interest, and to date, approximately one-third of the total target sample has been assessed and randomized into the intervention groups. We anticipate that recruitment will continue until March 2013, and it is estimated that all data collection will be complete by September 2013. The trial has been designed against the Consolidated Standards of Reporting Trials (CONSORT) reporting guidelines [28], and the results of the study are likely to form an evidence base for the feasibility of dementia-prevention campaigns to lead to lifestyle changes and the reduction of dementia risk factors at the population level.

The positive response to the study from individuals who traditionally do not see themselves as the target population for dementia-related studies, most of whom are still in the workforce, reflects community concern regarding dementia risk, and an interest in emerging prevention efforts. Therefore, successful outcomes of the current trial may be associated with actual public health impact upon making the intervention available at the population level. Although additional funds are likely to be required to update and improve aspects of the online intervention program, a particularly strong appeal of this intervention is the fully automated yet individually-tailored online delivery, with negligible maintenance costs.

The study has some limitations. The relatively small sample size limits the possibility of designing more than one control condition, and reduces the feasibility of meaningful long-term follow-up. The study will be underpowered to evaluate the effect (if any) of group within the BBL + FF condition, and this potentially limits the non-independence of the participants. Exploratory analyses of this effect will still be conducted. The project is, however, a pilot project, and the outcome data will form the basis for an application for funding for a larger scale trial. Finally, as is the case for other intervention trials recruiting healthy volunteers, the sample may not adequately represent the general population of those at risk of AD. However the inclusion criteria requiring three or more risk factors mitigates this to some extent. Ongoing efforts are being made to recruit individuals from diverse backgrounds.

## Conclusions

Interventions to prevent or delay the onset of clinical AD and other dementia are urgently needed, and global efforts are beginning to shift in this direction. The BBL trial provides a novel approach to addressing this pressing public health burden.

## Trial status

The trial is ongoing.

## Abbreviations

AD: Alzheimer’s disease; BBL: Body Brain Life; RCT: Randomized controlled trial.

## Competing interests

The authors declare that they have no competing interest.

## Authors’ contributions

All authors contributed to the design of the study and the drafting of the manuscript. KJA is the lead principal investigator and developed the study concept with NC. AB-F developed the behavior change framework for the intervention and is the project manager. GWR advised on the methodology and cognitive training components. PH advised on the medical information and clinical issues. All authors read and approved the final manuscript.

## References

[B1] Ayaori M Hisada T Yoshida H Shige H Ito T Nakajima K Higashi K Yonemura A Ishikawa T Ohsuzu F Effect of alcohol intake on the levels of plasma homocysteine in healthy males J Nutr Sci Vitaminol 2000 46 171 174 10.3177/jnsv.46.171 11185653

[B2] Barnes DE Yaffe K The projected effect of risk factor reduction on Alzheimer’s disease prevalence Lancet Neurol 2011 10 819 828 10.1016/S1474-4422(11)70072-2 21775213 PMC3647614

[B3] Fratiglioni L Qiu C Prevention of cognitive decline in ageing: dementia as the target, delayed onset as the goal Lancet Neurol 2011 10 778 779 10.1016/S1474-4422(11)70145-4 21775212

[B4] Anstey KJ Cherbuin N Budge M Young J Body mass index in midlife and late-life as a risk factor for dementia: a meta-analysis of prospective studies Obes Rev 2011 12 e426 e437 10.1111/j.1467-789X.2010.00825.x 21348917

[B5] Anstey KJ Lipnicki DM Low LF Cholesterol as a risk factor for dementia and cognitive decline: a systematic review of prospective studies with meta-analysis Am J Geriatr Psychiatry 2008 16 343 354 18448847 10.1097/JGP.0b013e31816b72d4

[B6] Anstey KJ Mack HA Cherbuin N Alcohol consumption as a risk factor for dementia and cognitive decline: meta-analysis of prospective studies Am J Geriatr Psychiatry 2009 17 542 555 10.1097/JGP.0b013e3181a2fd07 19546653

[B7] Anstey KJ von Sanden C Salim A O’Kearney R Smoking as a risk factor for dementia and cognitive decline: a meta-analysis of prospective studies Am J Epidemiol 2007 166 367 378 10.1093/aje/kwm116 17573335

[B8] Anstey KJ Cherbuin N Herath P Development of a new method for assessing global risk of alzheimer’s disease for use in population health approaches to prevention Prev Sci 2013 10.1007/s11121-012-0313-2 PMC3696462 23319292

[B9] Cherbuin N Reglade-Meslin C Kumar R Jacomb P Easteal S Christensen H Sachdev P Anstey KJ Risk factors of transition from normal cognition to mild cognitive disorder: the PATH through life study Dement Geriatr Cogn Disord 2009 28 47 55 10.1159/000229025 19628940

[B10] Sperling RA Aisen PS Beckett LA Bennett DA Craft S Fagan AM Iwatsubo T Jack CRJ Kaye J Wagster MV Phelps CH Toward defining the preclinical stages of Alzheimer’s disease: recommendations from the National Institute on Aging-Alzheimer’s Association workgroups on diagnostic guidelines for Alzheimer’s disease Alzheimers Dement 2011 7 280 292 10.1016/j.jalz.2011.03.003 21514248 PMC3220946

[B11] Hardeman W Kinmonth AL Michie S Sutton S Impact of a physical activity intervention program on cognitive predictors of behaviour among adults at risk of type 2 diabetes (ProActive randomised controlled trial) Int J Behav Nutr Phys Act 2009 6 16 10.1186/1479-5868-6-16 19292926 PMC2669044

[B12] Michie S Abraham C Interventions to change health behaviours: evidence-based or evidence-inspired? Psychol Health 2004 19 29 49 10.1080/0887044031000141199

[B13] Michie S van Stralen MM West R The behaviour change wheel: a new method for characterising and designing behaviour change interventions Implement Sci 2011 6 42 10.1186/1748-5908-6-42 21513547 PMC3096582

[B14] Cane J O’Connor D Michie S Validation of the theoretical domains framework for use in behaviour change and implementation research Implement Sci 2012 7 37 10.1186/1748-5908-7-37 22530986 PMC3483008

[B15] Michie S Abraham C Eccles MP Francis JJ Hardeman W Johnston M Strengthening evaluation and implementation by specifying components of behaviour change interventions: a study protocol Implement Sci 2011 6 10 10.1186/1748-5908-6-10 21299860 PMC3041694

[B16] Ahern DK Challenges and opportunities of eHealth research Am J Prev Med 2007 32 S75 S82 10.1016/j.amepre.2007.01.016 17466822

[B17] Noar SM Benac CN Harris MS Does tailoring matter? Meta-analytic review of tailored print health behavior change interventions Psychol Bull 2007 133 673 17592961 10.1037/0033-2909.133.4.673

[B18] Portnoy DB Scott-Sheldon LA Johnson BT Carey MP Computer-delivered interventions for health promotion and behavioral risk reduction: a meta-analysis of 75 randomized controlled trials, 1988–2007 Prev Med 2008 47 3 16 10.1016/j.ypmed.2008.02.014 18403003 PMC2572996

[B19] Riper H Spek V Boon B Conijn B Kramer J Martin-Abello K Smit F Effectiveness of E-self-help interventions for curbing adult problem drinking: a meta-analysis J Med Internet Res 2011 13 e42 10.2196/jmir.1691 21719411 PMC3221381

[B20] Muñoz RF Aguilera A Schueller SM Leykin Y Pérez-Stable EJ From online randomized controlled trials to participant preference studies: morphing the San Francisco stop smoking site into a worldwide smoking cessation resource J Med Internet Res 2012 14 e64 10.2196/jmir.1852 22739225 PMC3414852

[B21] Gatz M Reynolds CA John R Johansson B Mortimer JA Pedersen NL Telephone screening to identify potential dementia cases in a population-based sample of older adults Int Psychogeriatr 2002 14 273 289 10.1017/S1041610202008475 12475088

[B22] Ketola E Sipila R Makela M Effectiveness of individual lifestyle interventions in reducing cardiovascular disease and risk factors Ann Med 2000 32 239 251 10.3109/07853890009011767 10852140

[B23] Michie S Johnston M Abraham C Lawton R Parker D Walker A Making psychological theory useful for implementing evidence based practice: a consensus approach Qual Saf Health Care 2005 14 26 10.1136/qshc.2004.011155 15692000 PMC1743963

[B24] Low LF Anstey KJ Dementia literacy: recognition and beliefs on dementia of the Australian public Alzheimers Dement 2009 5 43 49 10.1016/j.jalz.2008.03.011 19118808

[B25] Fielding S Maclennan G Cook JA Ramsay CR A review of RCTs in four medical journals to assess the use of imputation to overcome missing data in quality of life outcomes Trials 2008 9 51 10.1186/1745-6215-9-51 18694492 PMC3225816

[B26] Elobeid MA Padilla MA McVie T Thomas O Brock DW Allison DB Missing data in randomized clinical trials for weight loss: scope of the problem, state of the field, and performance of statistical methods PLoS One 2009 4 e6624 10.1371/journal.pone.0006624 19675667 PMC2720539

[B27] Morrison LG Yardley L Powell J Michie S What design features are used in effective e-health interventions? A review using techniques from critical interpretive synthesis Telemed J E Health 2012 18 137 144 10.1089/tmj.2011.0062 22381060

[B28] Boutron I Moher D Altman DG Schulz KF Ravaud P Extending the CONSORT statement to randomized trials of nonpharmacologic treatment: explanation and elaboration Ann Intern Med 2008 148 295 18283207 10.7326/0003-4819-148-4-200802190-00008

